# A Compact Low-Frequency Acoustic Perfect Absorber Constructed with a Folded Slit

**DOI:** 10.3390/ma17235992

**Published:** 2024-12-06

**Authors:** Han Wang, Pengwei Ma, Xueling Fan

**Affiliations:** Xi’an Key Laboratory of Extreme Environment and Protection Technology, School of Aerospace Engineering, Xi’an Jiaotong University, Xi’an 710049, China

**Keywords:** perfect acoustic absorption, slit absorber, folded slit, tunable absorption, acoustic metamaterial

## Abstract

Tunable perfect acoustic absorption at subwavelength thickness has been a prominent topic in scientific research and engineering applications. Although metamaterials such as labyrinthine metasurfaces and coiling-up-space metamaterials can achieve subwavelength low-frequency acoustic absorption, efficiently realizing tunable absorption under uniform and limited size conditions remains challenging. In this paper, we introduce a folded slit to enhance the micro-slit acoustic absorber, effectively improving its low-frequency acoustic absorption performance and successfully achieving a perfect acoustic absorption coefficient of 0.99 at a thickness of only 3.1 cm. By adjusting just two parameters of the folded area, we can efficiently achieve a tunable resonant frequency ranging from 525 to 673 Hz and a tunable acoustic absorption bandwidth of 56.5% to 60.2%, simultaneously maintaining uniform external dimensions. Additionally, the folded-slit absorber demonstrates a broader acoustic absorption bandwidth at lower frequencies, enhancing broadband absorption capabilities in the low-frequency domain. These results hold significant potential for the design of highly efficient, thin and tunable acoustic absorbers.

## 1. Introduction

The presence of noise hinders individuals’ rest, study and work, leading to detrimental impacts on both human health and the surrounding environment [1]. With the development of industrial production, transportation and urban buildings, environmental noise is becoming increasingly serious. It has become a major public hazard polluting the human social environment [2,3]. In the field of aviation, aircraft engines produce intense noise during the service process, which greatly reduces the comfort of people in the aircraft [4]. Prolonged exposure to a noisy environment can have adverse effects on the health of the crew and passengers. Therefore, measures should be taken to mitigate noise effects and enhance aircraft comfort. This has important scientific and engineering application value and is also an important aspect of the future technological development of the aviation industry.

Conventional acoustic absorbers such as micro-slit absorbers [5,6], porous materials [7,8] and micro-perforated plate absorbers [9] exhibit a narrow acoustic absorption band, and their structural thickness is typically equivalent to a quarter of the working frequency wavelength [10]. Consequently, traditional absorbing materials/structures are thick when used to absorb noise, especially for low-frequency noise, and so cannot meet the technical requirements of compact-space noise reduction, such as for aircraft engines, automobiles and small rooms, at the present stage. For compact interior spaces, there are strict requirements for the volume and thickness of acoustic absorbers, which greatly limits the practical application potential of conventional absorbers. Therefore, it is very important to find a new structure with excellent acoustic absorption performance in the low-frequency range and the potential to reduce the thickness of the structural elements.

In recent years, acoustic metamaterials and metasurfaces have received a lot of attention and undergone rapid development because they present many excellent and special acoustic properties, enabling new methods and ideas for the innovation of aircraft engine noise reduction technology [11]. Acoustic absorption structures such as labyrinthine metasurfaces [12,13], folded-cavity metamaterials [14,15] and coiling-up-space metamaterials [16,17] can achieve low-frequency absorption under subwavelength thickness. However, because they have a large number of curved channels and a complex structure, it is difficult to achieve tunable acoustic absorption without changing their overall size. Therefore, it is difficult to maintain a consistent shape and achieve tunable and efficient acoustic absorption.

In the present study, we propose a novel design for an absorber with a folded slit. The folded slit was introduced by modifying the original micro-slit absorber design. The perfect acoustic absorption of the folded-slit absorber was investigated through simulations and experiments. Moreover, the folded-slit absorber efficiently achieved a tunable resonant frequency and a tunable acoustic absorption bandwidth by only adjusting the parameters of the folded area while maintaining uniform external dimensions. This approach may pave the way for highly efficient, thin and tunable acoustic absorbers.

## 2. Structure and Methods

### 2.1. Structure of Folded-Slit Absorber

**Figure 1:**
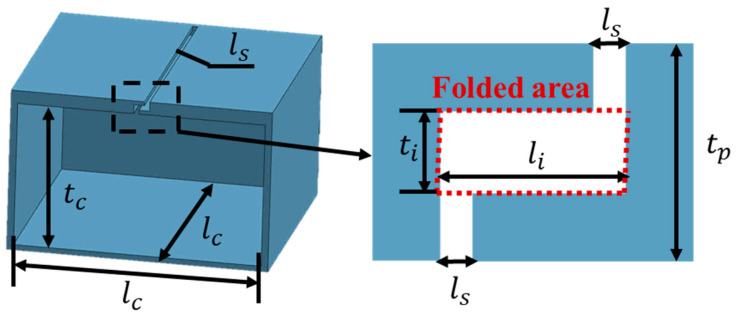
Structural diagram of the novel folded-slit absorber.

The novel folded-slit absorber consists of a folded-slit panel and a cavity, as illustrated in Figure 1. The parameter *t_p_* represents the thickness of the upper panel, while *t_c_* denotes the depth of the cavity. The parameter *l_c_* indicates the width of the cavity, and *l_s_* refers to the width of the slit. In the folded area, the thickness, *t_i_*, is *n* times the thickness of the upper panel, *t_p_* (where *n* = *t_i_*/*t_p_*, 0 < *n* < 1). Additionally, the width, *l_i_*, of this area is *m* times the width of the slit, *l_s_* (where *m* = *l_i_*/*l_s_*, 1 < *m* < *l_c_*/*l_s_*). By adjusting the parameters *m* and *n*, the dimensions of the folding area can be modified, thereby enhancing the ability to manipulate the acoustic impedance of the absorber.

### 2.2. Method of Finite Element Simulation

**Figure 2:**
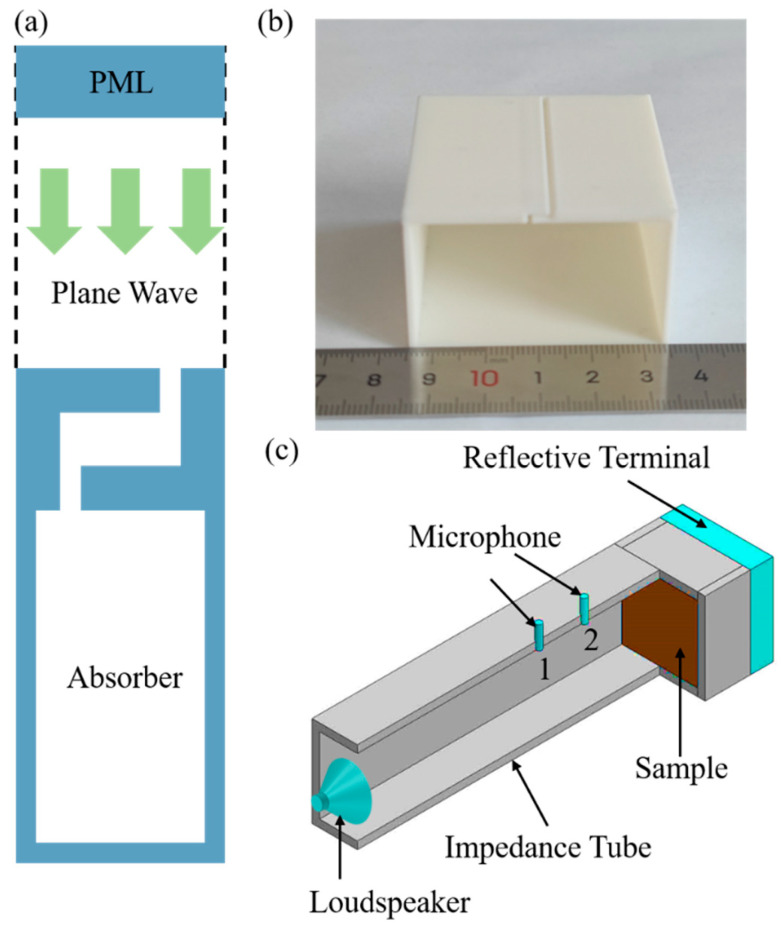
(**a**) The finite element model of the folded-slit absorber; (**b**) a photo of the folded-slit absorber; (**c**) a schematic illustration for the experimental realization of the designed acoustic absorbers.

The acoustic–thermoacoustic interaction models used to calculate the acoustic absorption coefficient were constructed using COMSOL 5.6 finite element software. As illustrated in Figure 2a, a plane wave is incident from above the absorber along a virtual impedance tube. The sound pressure of the plane wave, $P_{i},$ is given by

(1) $$P_{i}=Ae^{iky}$$

where A denotes the amplitude of acoustic pressure, and $k=\omega /c_{0}$ is the wavenumber. The pressure, $P_{y_{1}},\;P_{y_{2}},$ at the interface $y_{1}$, $y_{2}$ can be expressed as [18]

(2) $$P_{y_{1}}=A_{i}e^{iky_{1}}+A_{r}e^{-iky_{1}}$$

(3) $$P_{y_{2}}=A_{i}e^{iky_{2}}+A_{r}e^{-iky_{2}}$$

and is solved as

(4) $$A_{i}=\frac{b_{2}P_{y_{1}}-a_{2}P_{y_{2}}}{a_{1}b_{2}-a_{2}b_{1}},A_{r}=\frac{a_{1}P_{y_{2}}-b_{1}P_{y_{1}}}{a_{1}b_{2}-a_{2}b_{1}}$$

where $a_{1}=e^{iky_{1}}$, $a_{2}=e^{-iky_{1}}$, $b_{1}=e^{iky_{2}}$ and $b_{2}=e^{-iky_{2}}$. Thus, the absorption coefficient can be calculated by

(5) $$\alpha =1-{\left|\frac{A_{r}}{A_{i}}\right|}^{2}$$

A perfect matching layer (PML) was added to the top of the tube to avoid spurious refection. The wall of the structure was considered to be the sound hard boundary. The effects of viscous and heat transfer are included in the linearized Navier–Stokes equation. Boundary-layer meshes were applied in the finite element models due to the significant velocity and temperature gradients within the boundary layer. In the simulation, the following parameters were adopted: air density, $\rho_{0}$ = 1.21 $kg/m^{3}$; sound speed, $c_{0}=$ 343 m/s; dynamic viscosity, $\eta =1.56\times 10^{-5}Pa\cdot s$; temperature, T = 293.15 K; heat capacity at constant pressure, $C_{P}$ = 1.013 $\frac{kJ}{kg\cdot K};$ and thermal conductivity, $\mu =2.593\times 10^{-2}\;W/mk$.

### 2.3. Experimental Method

The folded-slit absorbers in this study were fabricated by a 3D printer (Creator-pro, FLASHFORGE, Hangzhou, China) with the fused deposition modeling (FDM) technique. Wire polylactic acid (PLA, FLASHFORGE, Hangzhou, China) was used as a raw material for manufacturing specimens of the folded-slit absorbers. The corresponding 3D printing parameters were as follows: a slice thickness of 0.12 mm, a nozzle preheating temperature of 200 °C, a printing speed of 50 mm/s, a printing accuracy of ±0.4 mm, etc. After the 3D printing process, the extra support materials were removed, and the specimens were cleaned manually. The printed sample is shown in Figure 2b.

The measurements of the absorption coefficient were performed using a square impedance tube system (F50, BSWA, Beijing, China), which complies with ASTM E1050-12 standards [19]. As shown in Figure 2c, a loudspeaker was mounted on one end of the tube, with the absorber mounted on the other end. The side length of the tube cross-section was 50 mm. The sound pressure was measured separately by two microphones with a distance interval of *s*:

(6) $$p_{1}=A_{1I}e^{jk_{0}x_{1}}+A_{1R}e^{-jk_{0}x_{1}}$$

(7) $$p_{2}=A_{2I}e^{jk_{0}x_{2}}+A_{2R}e^{-jk_{0}x_{2}}$$

where $A_{I}$ and $A_{R}$ are the sound pressure amplitudes of the incident wave and the reflected wave, respectively. Then, the transfer functions can be described as [20]

(8) $$H_{I}=\frac{A_{2I}}{A_{1I}}$$

(9) $$H_{R}=\frac{A_{2R}}{A_{1R}}$$

(10) $$H_{12}=\frac{p_{2}}{p_{1}}$$

The acoustic absorption coefficient measured in this experiment is

(11) $$\alpha =1-{\left|\frac{H_{12}-H_{I}}{H_{R}-H_{12}}e^{j2ks}\right|}^{2}$$

## 3. Results and Discussion

### 3.1. Low-Frequency Perfect Acoustic Absorption of the Folded-Slit Absorber

**Figure 3:**
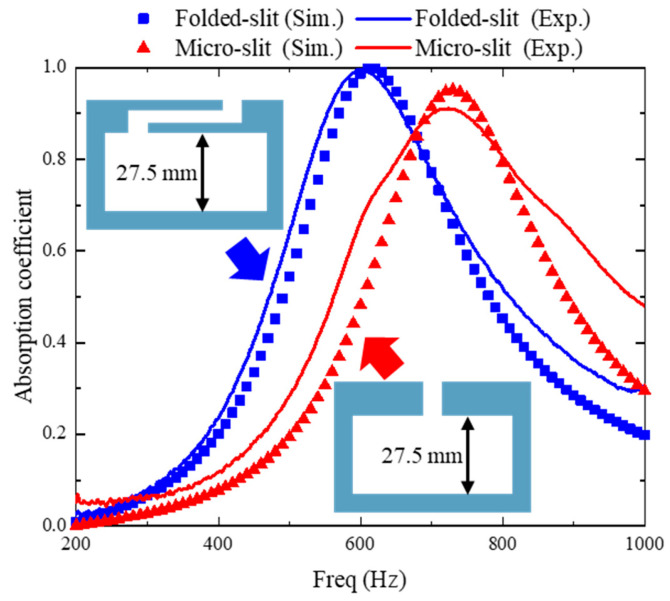
Performance comparison between the folded-slit absorber and micro-slit absorber.

Figure 3 compares the acoustic absorption coefficients of the folded-slit absorber with those of a traditional micro-slit absorber. The specific structural parameters for both types are detailed in Table 1. The experimental results indicate that the micro-slit absorber achieves its peak absorption at 721 Hz, with an absorption coefficient of 0.91. Notably, the cavity thickness of this absorber is 1/17 of the wavelength corresponding to the resonant frequency. In contrast, when the thickness and width of the folded-slit absorber are kept constant, its resonant frequency, *f_r_*, is measured to be 600 Hz, which is 16.7% lower than that of the micro-slit absorber. Furthermore, the cavity thickness of the folded-slit absorber is just 1/21 of the resonant frequency wavelength, but it attains a peak absorption coefficient of 1.0, signifying perfect acoustic absorption. These findings demonstrate that incorporating the folded slit effectively enhances low-frequency performance and facilitates the design of a perfect absorber at subwavelength scales.

In the simulation models, the structural walls were treated as hard sound boundaries. However, in reality, the actual material behaves as a lossy medium. This means that the sound energy loss was only approximated in the simulation models, potentially leading to discrepancies between the simulation and the measurement results. Nonetheless, a consistent conclusion can be drawn from comparing the simulation results: the folded-slit absorber achieves perfect acoustic absorption and lower-frequency absorption under uniform dimensions, exhibiting superior subwavelength performance.

**Figure 4:**
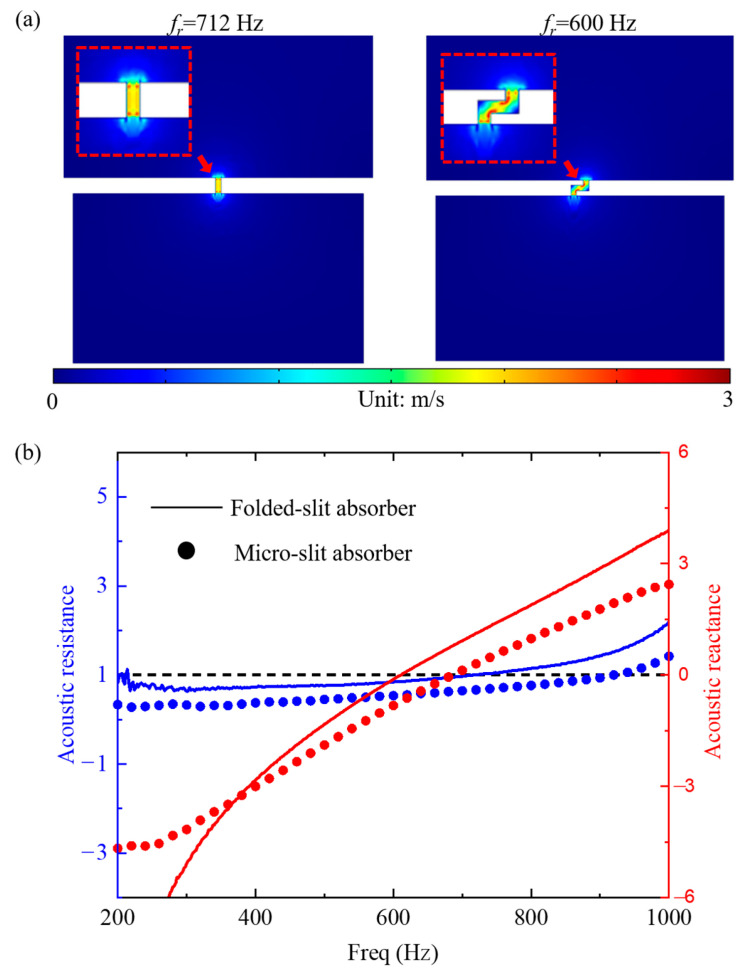
The absorption mechanism of the folded-slit absorber. (**a**) The particle velocities and (**b**) the acoustic resistance and reactance of the folded-slit and micro-slit absorbers at their resonant frequencies.

Figure 4a displays the air particle velocities of the folded-slit and micro-slit absorbers at their resonant frequencies. In both structures, the air particle velocities were significantly higher within the slits than elsewhere. At the resonant frequencies, the vigorous movement of air particles in the area formed by the slits resulted in peak velocities, akin to a resonant cavity dominated by sound mass. Conversely, the slit region of the micro-slit absorber is a straight channel, resulting in less intense particle vibration compared to the folded-slit absorber. The folded design prolongs the sound wave propagation path, increasing thermal viscosity loss due to the slits. Based on the relationship between the acoustic absorption coefficient and acoustic impedance,

(12) $$\alpha =1-{\left|\frac{Z_{s}-\rho_{0}c_{0}}{Z_{s}+\rho_{0}c_{0}}\right|}^{2},$$

when the acoustic reactance, Im(*Z_s_*), is 0 and the acoustic resistance, Re(*Z_s_*), approaches 1, the acoustic absorber demonstrates higher absorption efficiency. As shown in Figure 4b, the acoustic reactance of the micro-slit sound absorber nears 0 at the resonant frequency; however, the straight slit does not provide sufficient acoustic resistance, preventing perfect acoustic absorption. The folded-slit absorber enhances structural resistance, allowing for impedance matching at the resonance frequency, thus achieving perfect absorption. This further illustrates the effectiveness of employing folded slits in designing low-frequency acoustic absorbers.

### 3.2. Tunable Perfect Absorption of the Folded-Slit Absorber

When its overall dimensions are fixed, an absorber with tunable perfect acoustic absorption offers significant benefits for noise reduction across different contexts and facilitates the design of multi-unit coupled broadband acoustic absorption systems [21]. Therefore, a tunable perfect absorber is a crucial component for practical applications. Labyrinthine metasurfaces face challenges in achieving a tunable absorption bandwidth with a consistent shape due to the necessity of increasing the number of channels [12,13]. Conversely, coiling-up-space metamaterials [16,17] and folded-cavity metamaterials [14,15] can adjust their absorption frequencies while retaining the same dimensions. However, they involve complex bending channels and numerous structural parameters that complicate performance tuning. Thus, designing a tunable perfect absorber with constant dimensions remains a significant challenge. In the following, we demonstrate how folded-slit absorbers can be effectively tuned while maintaining a constant external shape by adjusting the folded slits.

**Figure 5:**
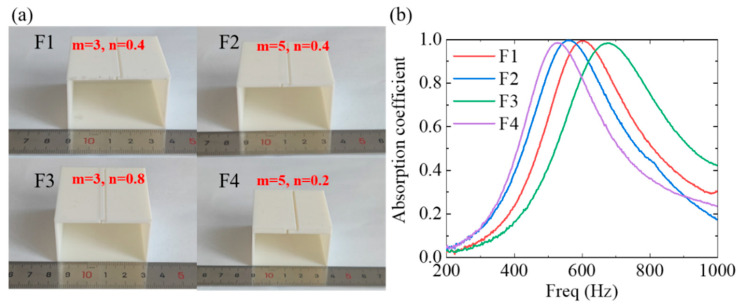
The tunable absorption of the folded-slit absorber. (**a**) Photos of the different samples; (**b**) the absorption coefficients of samples F1–F4.

Four samples with identical dimensions were fabricated, as depicted in Figure 5a. Each sample has a thickness of 31 mm and a width of 49.6 mm. The specific parameters are detailed in Table 2. Figure 5b illustrates a comparison of the absorption coefficients for the different samples. Notably, the folded area width, *l_i_*, of sample F2 is larger than that of sample F1. Sample F1 exhibits a resonant frequency of 600 Hz, while sample F2 has a resonant frequency of 561 Hz. These results indicate that increasing the ratio, *m*, of the folded area width, *l_i_*, to the slit width, *l_s_*, enhances the low-frequency performance of the structure.

In comparison to sample F3, sample F1 has a smaller folded area thickness, *t_i_*. The absorption coefficient comparison shows that the resonant frequency of sample F3 is 673 Hz, which represents a shift of 73 Hz to higher frequencies compared to sample F1. A smaller ratio, *n*, between the thickness, *t_i_*, of the folded area and the thickness, *t_p_*, of the upper panel facilitates low-frequency acoustic absorption in the folded-slit absorber.

For traditional micro-slit absorbers, achieving high absorption efficiency necessitates a thin upper panel thickness and a small slit width. When the slit width exceeds 1 mm, the acoustic absorption performance declines significantly, which leads to a demand for higher manufacturing precision and increases production costs [22,23]. The folded-slit absorber sample F4 designed in this study features a substantial panel thickness (5 mm) and slit width (1.5 mm) while maintaining an efficient absorption coefficient of greater than 0.98. The resonant frequency of sample F4 is 525 Hz, with its cavity thickness being only 1/26 of the resonant wavelength, thereby simplifying production and enhancing low-frequency performance. Additionally, sample F4 shares the same parameter m as sample F2, but with a smaller parameter n value, further indicating that a reduced n value promotes effective low-frequency acoustic absorption. The absorption bandwidth [21] of the acoustic absorber can be calculated as

(13) $$B=\frac{∆f}{f_{r}}$$

where ∆f represents the absorption band, with an acoustic absorption coefficient of greater than 0.5, and $f_{r}$ is the resonant frequency. The absorption bandwidths for samples F1 to F4 are 56.5%, 60.2%, 59.1% and 57.3%, respectively. In structures such as neck-extended absorbers, a lower resonant frequency typically correlates with a narrower absorption bandwidth under the constraints of perfect absorption and a constant external shape. However, in contrast to sample F1, samples F2 and F4 demonstrate a broader absorption bandwidth while achieving lower frequencies for perfect acoustic absorption, effectively overcoming this limitation.

Overall, the folded-slit absorber proposed in this study can efficiently achieve tunable perfect acoustic absorption under the conditions of constant external dimensions through merely adjusting the folded area parameters. It exhibits superior acoustic adjustment performance compared to previous studies on labyrinthine metasurfaces, coiling-up-space metamaterials, and folded-cavity metamaterials. This structure design lays the foundation for broadband absorption designs.

## 4. Conclusions

Achieving perfect low-frequency absorption with structures of subwavelength thickness poses a challenge for conventional acoustic absorbers. This paper presents the design of an acoustic absorber featuring a folded slit, demonstrating perfect absorption both through simulations and experiments. The conclusions are as follows:

- The folded-slit absorber achieves perfect acoustic absorption when the cavity thickness is only 1/21 of the resonant frequency’s wavelength. For the same dimensions, the resonant frequency is shifted down by 16.7% compared to traditional micro-slit absorbers, enhancing its performance in subwavelength low-frequency acoustic absorption.
- By adjusting the folded area parameters, *m* and *n*, the folded-slit absorber can achieve perfect acoustic absorption at multiple frequencies ranging from 525 to 673 Hz without altering its overall dimensions, demonstrating efficient tunability.
- With uniform dimensions, the acoustic absorption bandwidth broadens as the folded-slit absorber targets lower frequencies, facilitating the realization of broadband absorption in the low-frequency domain.

In summary, this paper proposes a compact folded-slit absorber that enhances the subwavelength low-frequency performance of micro-slit absorbers. With a thickness of only 3.1 cm, the folded-slit absorber efficiently achieves tunable perfect acoustic absorption. The proposed structure may contribute significantly to the design of highly efficient, ultrathin, and widely tunable acoustic absorbers, as well as to furthering broadband absorption design.

## Figures and Tables

**Table 1:** The structural parameters of the folded-slit absorber and micro-slit absorber.

Parameter	Folded-Slit Absorber	Micro-Slit Absorber	Unit
*t_p_*	2.5	2.5	mm
*t_c_*	27.5	27.5	mm
*l_c_*	47.0	47.0	mm
*l_s_*	1.0	1.0	mm
*t_i_*	1.0	/	mm
*l_i_*	3.0	/	mm

**Table 2:** The structure parameters of the samples.

Parameter	F1	F2	F3	F4	Unit
*t_p_*	2.5	2.5	2.5	5.0	mm
*t_c_*	27.5	27.5	27.5	25.0	mm
*l_c_*	47.0	47.0	47.0	47.0	mm
*l_s_*	1.0	1.0	1.0	1.5	mm
***t_i_***	1.0	1.0	2.0	1.0	mm
***l_i_***	3.0	5.0	3.0	7.5	mm
***m***	3.0	5.0	3.0	5.0	/
***n***	0.4	0.4	0.8	0.2	/

## Data Availability

The raw data supporting the conclusions of this article will be made available by the authors on request.
